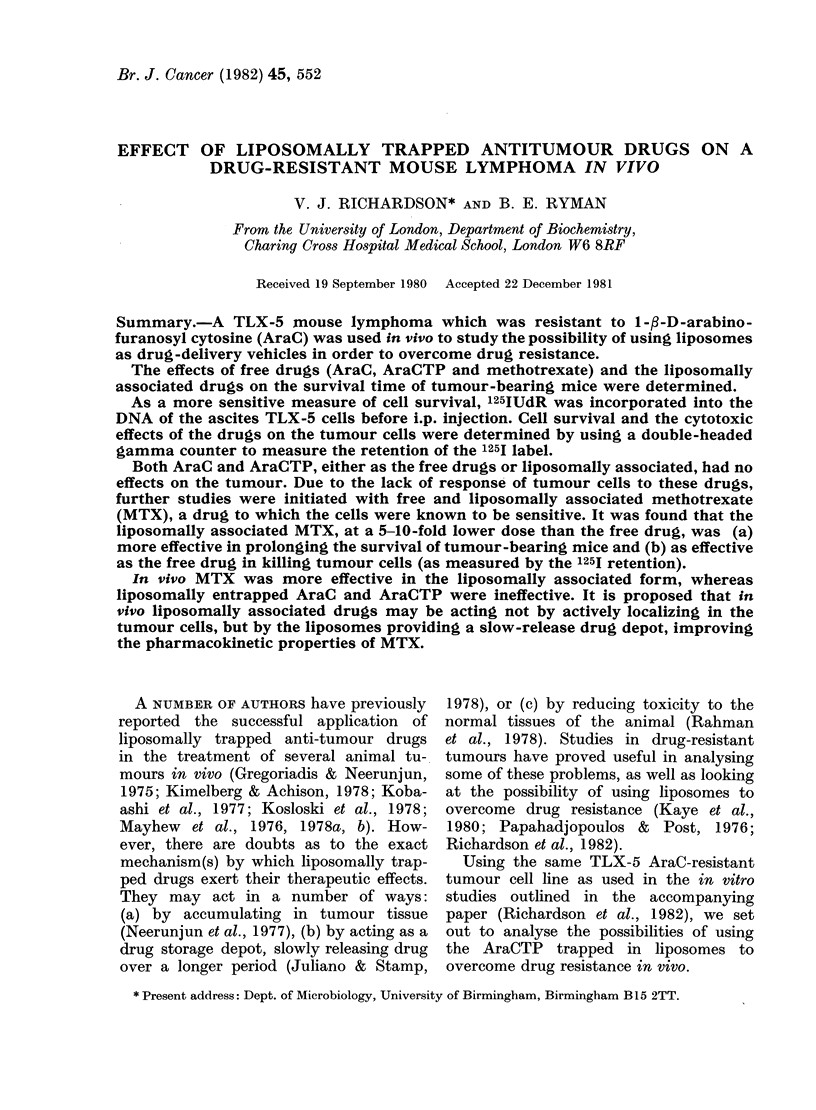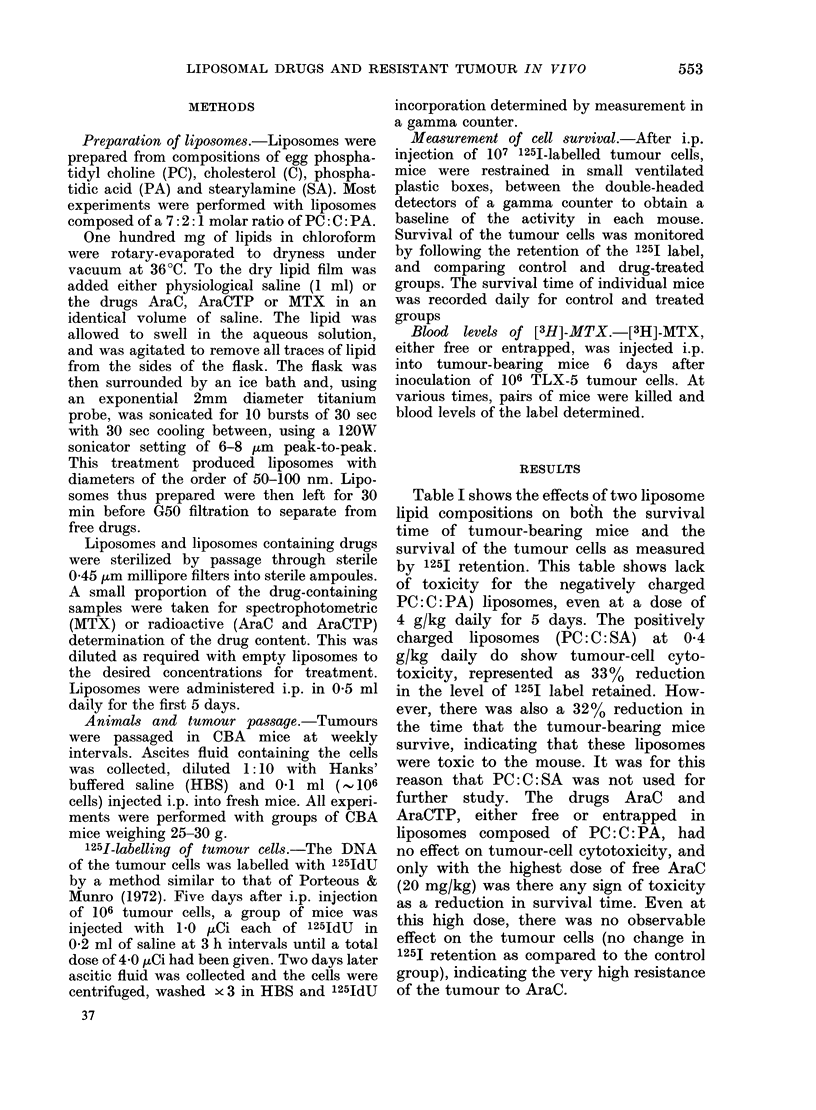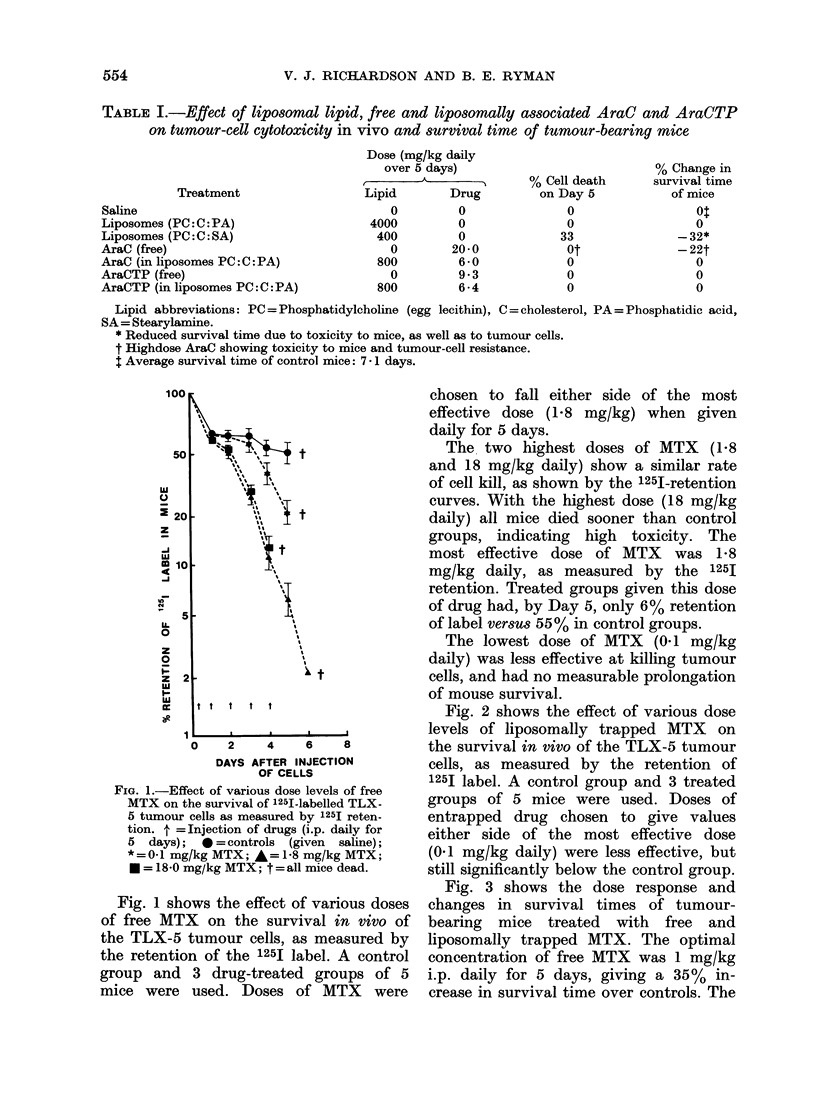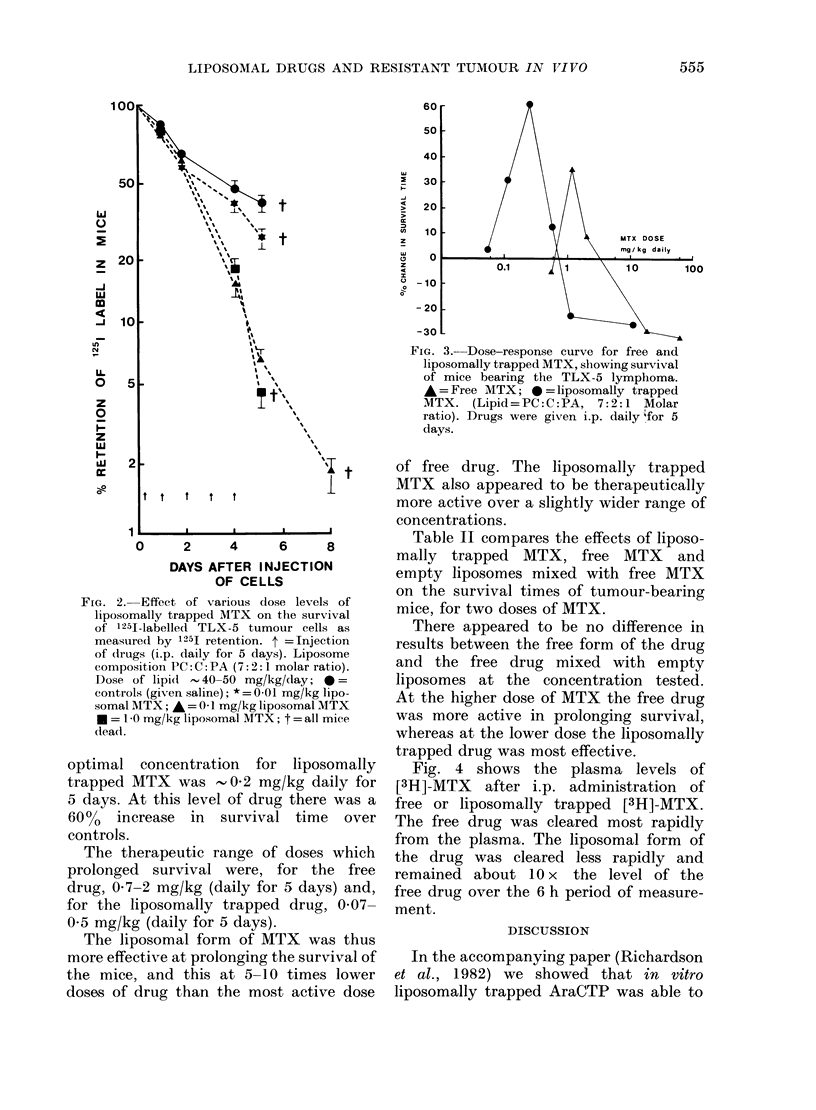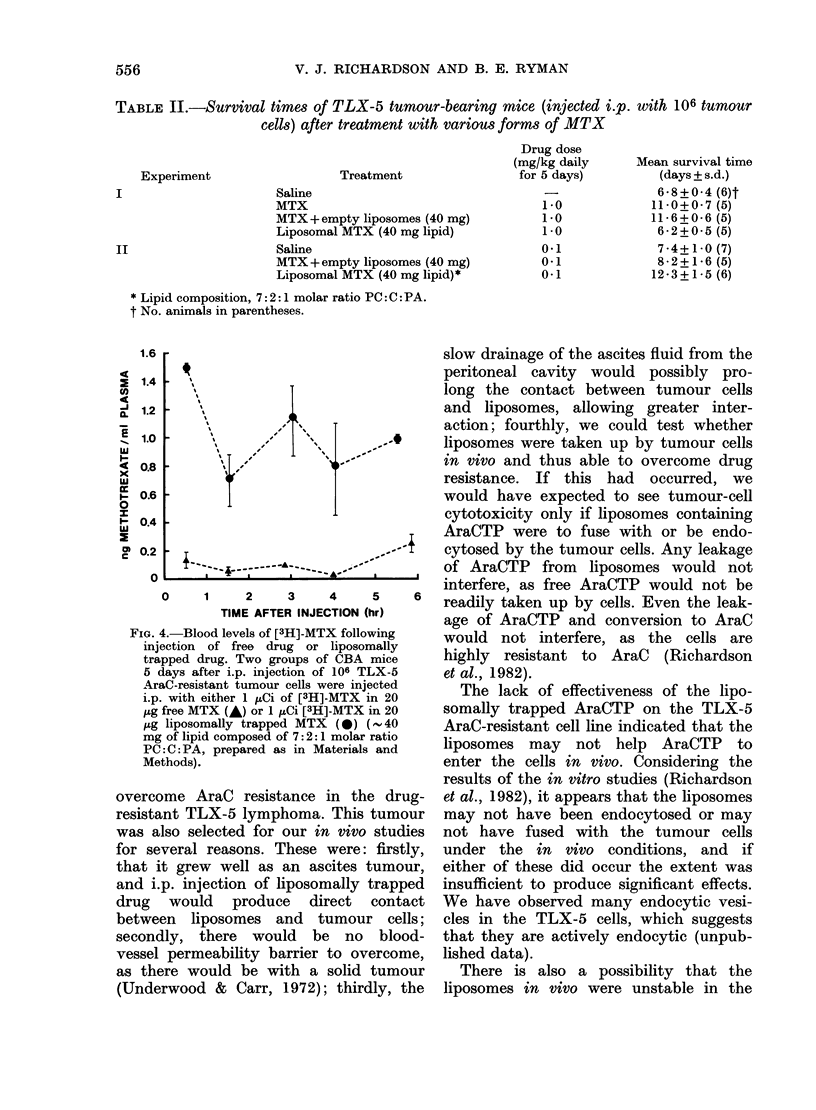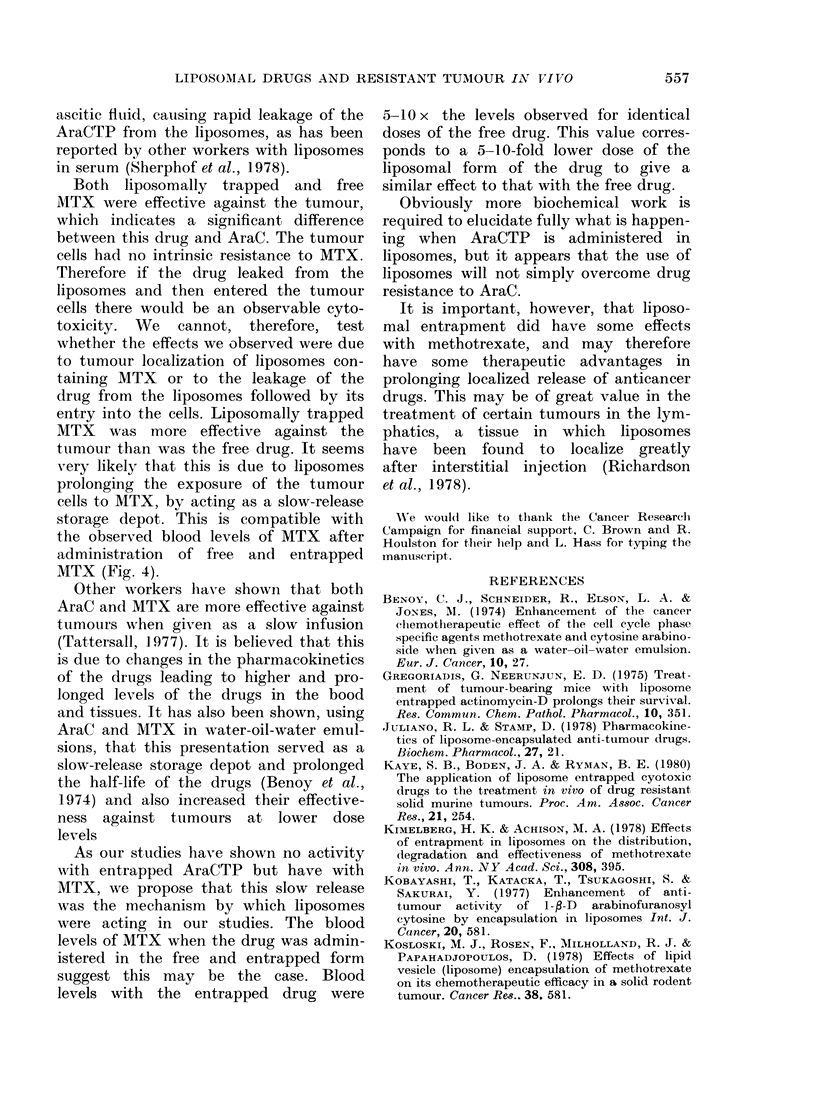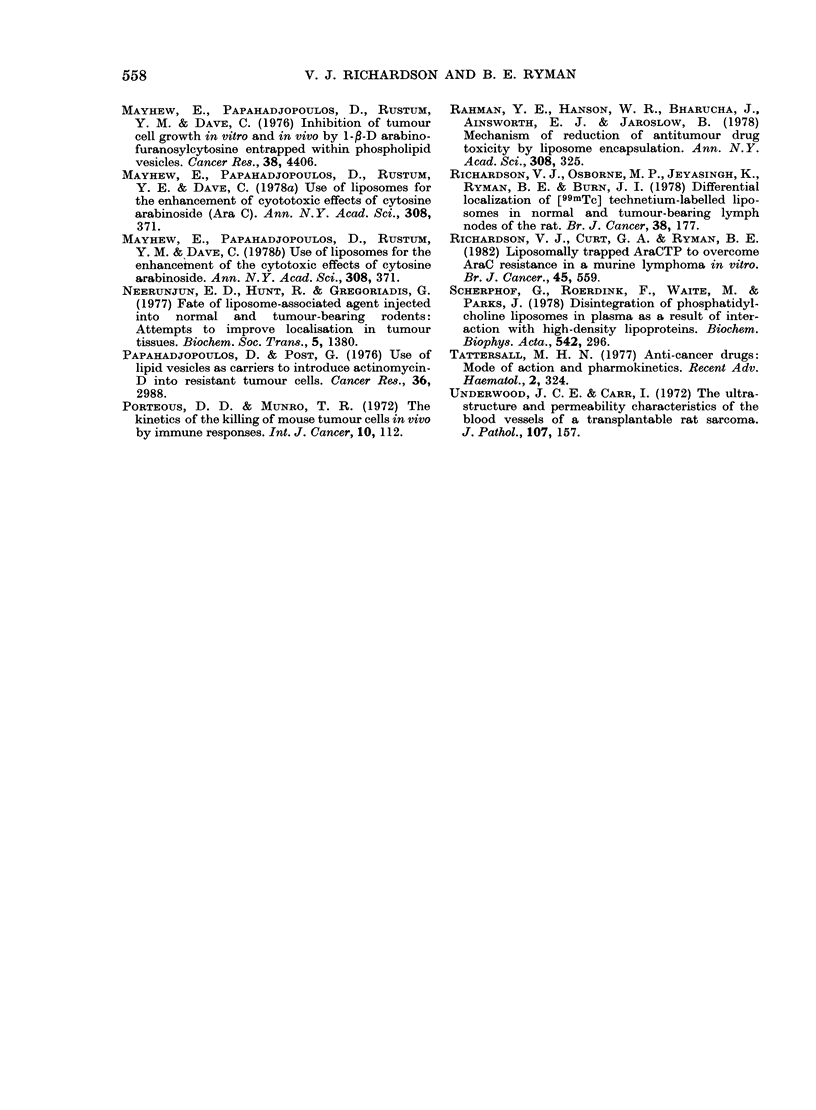# Effect of liposomally trapped antitumour drugs on a drug-resistant mouse lymphoma in vivo

**DOI:** 10.1038/bjc.1982.91

**Published:** 1982-04

**Authors:** V. J. Richardson, B. E. Ryman

## Abstract

A TLX-5 mouse lymphoma which was resistant to 1-β-D-arabinofuranosyl cytosine (AraC) was used *in vivo* to study the possibility of using liposomes as drug-delivery vehicles in order to overcome drug resistance.

The effects of free drugs (AraC, AraCTP and methotrexate) and the liposomally associated drugs on the survival time of tumour-bearing mice were determined.

As a more sensitive measure of cell survival, ^125^IUdR was incorporated into the DNA of the ascites TLX-5 cells before i.p. injection. Cell survival and the cytotoxic effects of the drugs on the tumour cells were determined by using a double-headed gamma counter to measure the retention of the ^125^I label.

Both AraC and AraCTP, either as the free drugs or liposomally associated, had no effects on the tumour. Due to the lack of response of tumour cells to these drugs, further studies were initiated with free and liposomally associated methotrexate (MTX), a drug to which the cells were known to be sensitive. It was found that the liposomally associated MTX, at a 5-10-fold lower dose than the free drug, was (a) more effective in prolonging the survival of tumour-bearing mice and (b) as effective as the free drug in killing tumour cells (as measured by the ^125^I retention).

***In vivo*** MTX was more effective in the liposomally associated form, whereas liposomally entrapped AraC and AraCTP were ineffective. It is proposed that *in vivo* liposomally associated drugs may be acting not by actively localizing in the tumour cells, but by the liposomes providing a slow-release drug depot, improving the pharmacokinetic properties of MTX.


*Br. J. Cancer* (1982) **45**, 552

### EFFECT OF LIPOSOMALLY TRAPPED ANTITUMOUR DRUGS ON A DRUG-RESISTANT MOUSE LYMPHOMA *IN VIVO*

V. J. RICHARDSON* AND B. E. RYMAN

*From the University of London, Department of Biochemistry, Charing Cross Hospital Medical School, London W6 8RF*

Received 19 September 1980 Accepted 22 December 1981

**Summary.—A TLX-5 mouse lymphoma which was resistant to 1-β-D-arabinofuranosyl cytosine (AraC) was used *in vivo* to study the possibility of using liposomes as drug-delivery vehicles in order to overcome drug resistance.**

**The effects of free drugs (AraC, AraCTP and methotrexate) and the liposomally associated drugs on the survival time of tumour-bearing mice were determined.**

**As a more sensitive measure of cell survival, $^{125}$IUdR was incorporated into the DNA of the ascites TLX-5 cells before i.p. injection. Cell survival and the cytotoxic effects of the drugs on the tumour cells were determined by using a double-headed gamma counter to measure the retention of the $^{125}$I label.**

**Both AraC and AraCTP, either as the free drugs or liposomally associated, had no effects on the tumour. Due to the lack of response of tumour cells to these drugs, further studies were initiated with free and liposomally associated methotrexate (MTX), a drug to which the cells were known to be sensitive. It was found that the liposomally associated MTX, at a 5–10-fold lower dose than the free drug, was (a) more effective in prolonging the survival of tumour-bearing mice and (b) as effective as the free drug in killing tumour cells (as measured by the $^{125}$I retention).**

***In vivo* MTX was more effective in the liposomally associated form, whereas liposomally entrapped AraC and AraCTP were ineffective. It is proposed that *in vivo* liposomally associated drugs may be acting not by actively localizing in the tumour cells, but by the liposomes providing a slow-release drug depot, improving the pharmacokinetic properties of MTX.**

A NUMBER OF AUTHORS have previously reported the successful application of liposomally trapped anti-tumour drugs in the treatment of several animal tumours *in vivo* (Gregoriadis & Neerunjun, 1975; Kimelberg & Achison, 1978; Kobaashi *et al.*, 1977; Kosloski *et al.*, 1978; Mayhew *et al.*, 1976, 1978*a*, *b*). However, there are doubts as to the exact mechanism(s) by which liposomally trapped drugs exert their therapeutic effects. They may act in a number of ways: (a) by accumulating in tumour tissue (Neerunjun *et al.*, 1977), (b) by acting as a drug storage depot, slowly releasing drug over a longer period (Juliano & Stamp, 1978), or (c) by reducing toxicity to the normal tissues of the animal (Rahman *et al.*, 1978). Studies in drug-resistant tumours have proved useful in analysing some of these problems, as well as looking at the possibility of using liposomes to overcome drug resistance (Kaye *et al.*, 1980; Papahadjopoulos & Post, 1976; Richardson *et al.*, 1982).

Using the same TLX-5 AraC-resistant tumour cell line as used in the *in vitro* studies outlined in the accompanying paper (Richardson *et al.*, 1982), we set out to analyse the possibilities of using the AraCTP trapped in liposomes to overcome drug resistance *in vivo*.

* Present address: Dept. of Microbiology, University of Birmingham, Birmingham B15 2TT.

### METHODS

*Preparation of liposomes.*—Liposomes were prepared from compositions of egg phosphatidyl choline (PC), cholesterol (C), phosphatidic acid (PA) and stearylamine (SA). Most experiments were performed with liposomes composed of a 7:2:1 molar ratio of PC:C:PA.

One hundred mg of lipids in chloroform were rotary-evaporated to dryness under vacuum at 36°C. To the dry lipid film was added either physiological saline (1 ml) or the drugs AraC, AraCTP or MTX in an identical volume of saline. The lipid was allowed to swell in the aqueous solution, and was agitated to remove all traces of lipid from the sides of the flask. The flask was then surrounded by an ice bath and, using an exponential 2mm diameter titanium probe, was sonicated for 10 bursts of 30 sec with 30 sec cooling between, using a 120W sonicator setting of 6–8 $\mu$m peak-to-peak. This treatment produced liposomes with diameters of the order of 50–100 nm. Liposomes thus prepared were then left for 30 min before G50 filtration to separate from free drugs.

Liposomes and liposomes containing drugs were sterilized by passage through sterile 0·45 $\mu$m millipore filters into sterile ampoules. A small proportion of the drug-containing samples were taken for spectrophotometric (MTX) or radioactive (AraC and AraCTP) determination of the drug content. This was diluted as required with empty liposomes to the desired concentrations for treatment. Liposomes were administered i.p. in 0·5 ml daily for the first 5 days.

*Animals and tumour passage.*—Tumours were passaged in CBA mice at weekly intervals. Ascites fluid containing the cells was collected, diluted 1:10 with Hanks' buffered saline (HBS) and 0·1 ml ($\sim 10^6$ cells) injected i.p. into fresh mice. All experiments were performed with groups of CBA mice weighing 25–30 g.

*$^{125}$I-labelling of tumour cells.*—The DNA of the tumour cells was labelled with $^{125}$IdU by a method similar to that of Porteous & Munro (1972). Five days after i.p. injection of $10^6$ tumour cells, a group of mice was injected with 1·0 $\mu$Ci each of $^{125}$IdU in 0·2 ml of saline at 3 h intervals until a total dose of 4·0 $\mu$Ci had been given. Two days later ascitic fluid was collected and the cells were centrifuged, washed $\times 3$ in HBS and $^{125}$IdU incorporation determined by measurement in a gamma counter.

*Measurement of cell survival.*—After i.p. injection of $10^7$ $^{125}$I-labelled tumour cells, mice were restrained in small ventilated plastic boxes, between the double-headed detectors of a gamma counter to obtain a baseline of the activity in each mouse. Survival of the tumour cells was monitored by following the retention of the $^{125}$I label, and comparing control and drug-treated groups. The survival time of individual mice was recorded daily for control and treated groups

*Blood levels of [$^3$H]-MTX.*—[$^3$H]-MTX, either free or entrapped, was injected i.p. into tumour-bearing mice 6 days after inoculation of $10^6$ TLX-5 tumour cells. At various times, pairs of mice were killed and blood levels of the label determined.

### RESULTS

Table I shows the effects of two liposome lipid compositions on both the survival time of tumour-bearing mice and the survival of the tumour cells as measured by $^{125}$I retention. This table shows lack of toxicity for the negatively charged PC:C:PA) liposomes, even at a dose of 4 g/kg daily for 5 days. The positively charged liposomes (PC:C:SA) at 0·4 g/kg daily do show tumour-cell cytotoxicity, represented as 33% reduction in the level of $^{125}$I label retained. However, there was also a 32% reduction in the time that the tumour-bearing mice survive, indicating that these liposomes were toxic to the mouse. It was for this reason that PC:C:SA was not used for further study. The drugs AraC and AraCTP, either free or entrapped in liposomes composed of PC:C:PA, had no effect on tumour-cell cytotoxicity, and only with the highest dose of free AraC (20 mg/kg) was there any sign of toxicity as a reduction in survival time. Even at this high dose, there was no observable effect on the tumour cells (no change in $^{125}$I retention as compared to the control group), indicating the very high resistance of the tumour to AraC.

TABLE I.—*Effect of liposomal lipid, free and liposomally associated AraC and AraCTP on tumour-cell cytotoxicity* in vivo *and survival time of tumour-bearing mice*

| Treatment | Dose (mg/kg daily over 5 days) Lipid | Drug | % Cell death on Day 5 | % Change in survival time of mice |
|---|---|---|---|---|
| Saline | 0 | 0 | 0 | 0‡ |
| Liposomes (PC:C:PA) | 4000 | 0 | 0 | 0 |
| Liposomes (PC:C:SA) | 400 | 0 | 33 | −32* |
| AraC (free) | 0 | 20·0 | 0† | −22† |
| AraC (in liposomes PC:C:PA) | 800 | 6·0 | 0 | 0 |
| AraCTP (free) | 0 | 9·3 | 0 | 0 |
| AraCTP (in liposomes PC:C:PA) | 800 | 6·4 | 0 | 0 |

Lipid abbreviations: PC = Phosphatidylcholine (egg lecithin), C = cholesterol, PA = Phosphatidic acid, SA = Stearylamine.

* Reduced survival time due to toxicity to mice, as well as to tumour cells.

† Highdose AraC showing toxicity to mice and tumour-cell resistance.

‡ Average survival time of control mice: 7·1 days.

FIG. 1.—Effect of various dose levels of free MTX on the survival of $^{125}$I-labelled TLX-5 tumour cells as measured by $^{125}$I retention. ↑ = Injection of drugs (i.p. daily for 5 days); ● = controls (given saline); * = 0·1 mg/kg MTX; ▲ = 1·8 mg/kg MTX; ■ = 18·0 mg/kg MTX; † = all mice dead.

Fig. 1 shows the effect of various doses of free MTX on the survival *in vivo* of the TLX-5 tumour cells, as measured by the retention of the $^{125}$I label. A control group and 3 drug-treated groups of 5 mice were used. Doses of MTX were chosen to fall either side of the most effective dose (1·8 mg/kg) when given daily for 5 days.

The two highest doses of MTX (1·8 and 18 mg/kg daily) show a similar rate of cell kill, as shown by the $^{125}$I-retention curves. With the highest dose (18 mg/kg daily) all mice died sooner than control groups, indicating high toxicity. The most effective dose of MTX was 1·8 mg/kg daily, as measured by the $^{125}$I retention. Treated groups given this dose of drug had, by Day 5, only 6% retention of label *versus* 55% in control groups.

The lowest dose of MTX (0·1 mg/kg daily) was less effective at killing tumour cells, and had no measurable prolongation of mouse survival.

Fig. 2 shows the effect of various dose levels of liposomally trapped MTX on the survival *in vivo* of the TLX-5 tumour cells, as measured by the retention of $^{125}$I label. A control group and 3 treated groups of 5 mice were used. Doses of entrapped drug chosen to give values either side of the most effective dose (0·1 mg/kg daily) were less effective, but still significantly below the control group.

Fig. 3 shows the dose response and changes in survival times of tumour-bearing mice treated with free and liposomally trapped MTX. The optimal concentration of free MTX was 1 mg/kg i.p. daily for 5 days, giving a 35% increase in survival time over controls. The

FIG. 2.—Effect of various dose levels of liposomally trapped MTX on the survival of $^{125}$I-labelled TLX-5 tumour cells as measured by $^{125}$I retention. ↑ = Injection of drugs (i.p. daily for 5 days). Liposome composition PC:C:PA (7:2:1 molar ratio). Dose of lipid ~40–50 mg/kg/day; ● = controls (given saline); ★ = 0·01 mg/kg liposomal MTX; ▲ = 0·1 mg/kg liposomal MTX ■ = 1·0 mg/kg liposomal MTX; † = all mice dead.

FIG. 3.—Dose–response curve for free and liposomally trapped MTX, showing survival of mice bearing the TLX-5 lymphoma. ▲ = Free MTX; ● = liposomally trapped MTX. (Lipid = PC:C:PA, 7:2:1 Molar ratio). Drugs were given i.p. daily for 5 days.

optimal concentration for liposomally trapped MTX was ~0·2 mg/kg daily for 5 days. At this level of drug there was a 60% increase in survival time over controls.

The therapeutic range of doses which prolonged survival were, for the free drug, 0·7–2 mg/kg (daily for 5 days) and, for the liposomally trapped drug, 0·07–0·5 mg/kg (daily for 5 days).

The liposomal form of MTX was thus more effective at prolonging the survival of the mice, and this at 5–10 times lower doses of drug than the most active dose of free drug. The liposomally trapped MTX also appeared to be therapeutically more active over a slightly wider range of concentrations.

Table II compares the effects of liposomally trapped MTX, free MTX and empty liposomes mixed with free MTX on the survival times of tumour-bearing mice, for two doses of MTX.

There appeared to be no difference in results between the free form of the drug and the free drug mixed with empty liposomes at the concentration tested. At the higher dose of MTX the free drug was more active in prolonging survival, whereas at the lower dose the liposomally trapped drug was most effective.

Fig. 4 shows the plasma levels of [$^3$H]-MTX after i.p. administration of free or liposomally trapped [$^3$H]-MTX. The free drug was cleared most rapidly from the plasma. The liposomal form of the drug was cleared less rapidly and remained about 10× the level of the free drug over the 6 h period of measurement.

### DISCUSSION

In the accompanying paper (Richardson *et al.*, 1982) we showed that *in vitro* liposomally trapped AraCTP was able to

TABLE II.—*Survival times of TLX-5 tumour-bearing mice (injected i.p. with* $10^6$ *tumour cells) after treatment with various forms of MTX*

| Experiment | Treatment | Drug dose (mg/kg daily for 5 days) | Mean survival time (days ± s.d.) |
|---|---|---|---|
| I | Saline | — | 6·8±0·4 (6)† |
| | MTX | 1·0 | 11·0±0·7 (5) |
| | MTX+empty liposomes (40 mg) | 1·0 | 11·6±0·6 (5) |
| | Liposomal MTX (40 mg lipid) | 1·0 | 6·2±0·5 (5) |
| II | Saline | 0·1 | 7·4±1·0 (7) |
| | MTX+empty liposomes (40 mg) | 0·1 | 8·2±1·6 (5) |
| | Liposomal MTX (40 mg lipid)* | 0·1 | 12·3±1·5 (6) |

* Lipid composition, 7:2:1 molar ratio PC:C:PA.
† No. animals in parentheses.

FIG. 4.—Blood levels of [$^3$H]-MTX following injection of free drug or liposomally trapped drug. Two groups of CBA mice 5 days after i.p. injection of $10^6$ TLX-5 AraC-resistant tumour cells were injected i.p. with either 1 $\mu$Ci of [$^3$H]-MTX in 20 $\mu$g free MTX (▲) or 1 $\mu$Ci [$^3$H]-MTX in 20 $\mu$g liposomally trapped MTX (●) (~40 mg of lipid composed of 7:2:1 molar ratio PC:C:PA, prepared as in Materials and Methods).

overcome AraC resistance in the drug-resistant TLX-5 lymphoma. This tumour was also selected for our *in vivo* studies for several reasons. These were: firstly, that it grew well as an ascites tumour, and i.p. injection of liposomally trapped drug would produce direct contact between liposomes and tumour cells; secondly, there would be no blood-vessel permeability barrier to overcome, as there would be with a solid tumour (Underwood & Carr, 1972); thirdly, the slow drainage of the ascites fluid from the peritoneal cavity would possibly prolong the contact between tumour cells and liposomes, allowing greater interaction; fourthly, we could test whether liposomes were taken up by tumour cells *in vivo* and thus able to overcome drug resistance. If this had occurred, we would have expected to see tumour-cell cytotoxicity only if liposomes containing AraCTP were to fuse with or be endocytosed by the tumour cells. Any leakage of AraCTP from liposomes would not interfere, as free AraCTP would not be readily taken up by cells. Even the leakage of AraCTP and conversion to AraC would not interfere, as the cells are highly resistant to AraC (Richardson *et al.*, 1982).

The lack of effectiveness of the liposomally trapped AraCTP on the TLX-5 AraC-resistant cell line indicated that the liposomes may not help AraCTP to enter the cells *in vivo*. Considering the results of the *in vitro* studies (Richardson *et al.*, 1982), it appears that the liposomes may not have been endocytosed or may not have fused with the tumour cells under the *in vivo* conditions, and if either of these did occur the extent was insufficient to produce significant effects. We have observed many endocytic vesicles in the TLX-5 cells, which suggests that they are actively endocytic (unpublished data).

There is also a possibility that the liposomes *in vivo* were unstable in the ascitic fluid, causing rapid leakage of the AraCTP from the liposomes, as has been reported by other workers with liposomes in serum (Sherphof *et al.*, 1978).

Both liposomally trapped and free MTX were effective against the tumour, which indicates a significant difference between this drug and AraC. The tumour cells had no intrinsic resistance to MTX. Therefore if the drug leaked from the liposomes and then entered the tumour cells there would be an observable cytotoxicity. We cannot, therefore, test whether the effects we observed were due to tumour localization of liposomes containing MTX or to the leakage of the drug from the liposomes followed by its entry into the cells. Liposomally trapped MTX was more effective against the tumour than was the free drug. It seems very likely that this is due to liposomes prolonging the exposure of the tumour cells to MTX, by acting as a slow-release storage depot. This is compatible with the observed blood levels of MTX after administration of free and entrapped MTX (Fig. 4).

Other workers have shown that both AraC and MTX are more effective against tumours when given as a slow infusion (Tattersall, 1977). It is believed that this is due to changes in the pharmacokinetics of the drugs leading to higher and prolonged levels of the drugs in the bood and tissues. It has also been shown, using AraC and MTX in water-oil-water emulsions, that this presentation served as a slow-release storage depot and prolonged the half-life of the drugs (Benoy *et al.*, 1974) and also increased their effectiveness against tumours at lower dose levels

As our studies have shown no activity with entrapped AraCTP but have with MTX, we propose that this slow release was the mechanism by which liposomes were acting in our studies. The blood levels of MTX when the drug was administered in the free and entrapped form suggest this may be the case. Blood levels with the entrapped drug were 5–10 × the levels observed for identical doses of the free drug. This value corresponds to a 5–10-fold lower dose of the liposomal form of the drug to give a similar effect to that with the free drug.

Obviously more biochemical work is required to elucidate fully what is happening when AraCTP is administered in liposomes, but it appears that the use of liposomes will not simply overcome drug resistance to AraC.

It is important, however, that liposomal entrapment did have some effects with methotrexate, and may therefore have some therapeutic advantages in prolonging localized release of anticancer drugs. This may be of great value in the treatment of certain tumours in the lymphatics, a tissue in which liposomes have been found to localize greatly after interstitial injection (Richardson *et al.*, 1978).

We would like to thank the Cancer Research Campaign for financial support, C. Brown and R. Houlston for their help and L. Hass for typing the manuscript.

### REFERENCES

BENOY, C. J., SCHNEIDER, R., ELSON, L. A. & JONES, M. (1974) Enhancement of the cancer chemotherapeutic effect of the cell cycle phase specific agents methotrexate and cytosine arabinoside when given as a water–oil–water emulsion. *Eur. J. Cancer*, **10**, 27.

GREGORIADIS, G. NEERUNJUN, E. D. (1975) Treatment of tumour-bearing mice with liposome entrapped actinomycin-D prolongs their survival. *Res. Commun. Chem. Pathol. Pharmacol.*, **10**, 351.

JULIANO, R. L. & STAMP, D. (1978) Pharmacokinetics of liposome-encapsulated anti-tumour drugs. *Biochem. Pharmacol.*, **27**, 21.

KAYE, S. B., BODEN, J. A. & RYMAN, B. E. (1980) The application of liposome entrapped cyotoxic drugs to the treatment *in vivo* of drug resistant solid murine tumours. *Proc. Am. Assoc. Cancer Res.*, **21**, 254.

KIMELBERG, H. K. & ACHISON, M. A. (1978) Effects of entrapment in liposomes on the distribution, degradation and effectiveness of methotrexate *in vivo*. *Ann. NY Acad. Sci.*, **308**, 395.

KOBAYASHI, T., KATACKA, T., TSUKAGOSHI, S. & SAKURAI, Y. (1977) Enhancement of antitumour activity of 1-$\beta$-D arabinofuranosyl cytosine by encapsulation in liposomes *Int. J. Cancer*, **20**, 581.

KOSLOSKI, M. J., ROSEN, F., MILHOLLAND, R. J. & PAPAHADJOPOULOS, D. (1978) Effects of lipid vesicle (liposome) encapsulation of methotrexate on its chemotherapeutic efficacy in a solid rodent tumour. *Cancer Res.*. **38**, 581.

MAYHEW, E., PAPAHADJOPOULOS, D., RUSTUM, Y. M. & DAVE, C. (1976) Inhibition of tumour cell growth *in vitro* and *in vivo* by 1-β-D arabinofuranosylcytosine entrapped within phospholipid vesicles. *Cancer Res.*, **38**, 4406.

MAYHEW, E., PAPAHADJOPOULOS, D., RUSTUM, Y. E. & DAVE, C. (1978*a*) Use of liposomes for the enhancement of cyototoxic effects of cytosine arabinoside (Ara C). *Ann. N.Y. Acad. Sci.*, **308**, 371.

MAYHEW, E., PAPAHADJOPOULOS, D., RUSTUM, Y. M. & DAVE, C. (1978*b*) Use of liposomes for the enhancement of the cytotoxic effects of cytosine arabinoside. *Ann. N.Y. Acad. Sci.*, **308**, 371.

NEERUNJUN, E. D., HUNT, R. & GREGORIADIS, G. (1977) Fate of liposome-associated agent injected into normal and tumour-bearing rodents: Attempts to improve localisation in tumour tissues. *Biochem. Soc. Trans.*, **5**, 1380.

PAPAHADJOPOULOS, D. & POST, G. (1976) Use of lipid vesicles as carriers to introduce actinomycin-D into resistant tumour cells. *Cancer Res.*, **36**, 2988.

PORTEOUS, D. D. & MUNRO, T. R. (1972) The kinetics of the killing of mouse tumour cells *in vivo* by immune responses. *Int. J. Cancer*, **10**, 112.

RAHMAN, Y. E., HANSON, W. R., BHARUCHA, J., AINSWORTH, E. J. & JAROSLOW, B. (1978) Mechanism of reduction of antitumour drug toxicity by liposome encapsulation. *Ann. N.Y. Acad. Sci.*, **308**, 325.

RICHARDSON, V. J., OSBORNE, M. P., JEYASINGH, K., RYMAN, B. E. & BURN, J. I. (1978) Differential localization of [$^{99m}$Tc] technetium-labelled liposomes in normal and tumour-bearing lymph nodes of the rat. *Br. J. Cancer*, **38**, 177.

RICHARDSON, V. J., CURT, G. A. & RYMAN, B. E. (1982) Liposomally trapped AraCTP to overcome AraC resistance in a murine lymphoma *in vitro*. *Br. J. Cancer.*, **45**, 559.

SCHERPHOF, G., ROERDINK, F., WAITE, M. & PARKS, J. (1978) Disintegration of phosphatidylcholine liposomes in plasma as a result of interaction with high-density lipoproteins. *Biochem. Biophys. Acta.*, **542**, 296.

TATTERSALL, M. H. N. (1977) Anti-cancer drugs: Mode of action and pharmokinetics. *Recent Adv. Haematol.*, **2**, 324.

UNDERWOOD, J. C. E. & CARR, I. (1972) The ultrastructure and permeability characteristics of the blood vessels of a transplantable rat sarcoma. *J. Pathol.*, **107**, 157.